# Changes in physio-biochemical parameters and expression of metallothioneins in *Avena sativa* L. in response to drought

**DOI:** 10.1038/s41598-023-29394-2

**Published:** 2023-02-12

**Authors:** Wiktoria Konieczna, Marzena Warchoł, Agnieszka Mierek-Adamska, Edyta Skrzypek, Piotr Waligórski, Agnieszka Piernik, Grażyna B. Dąbrowska

**Affiliations:** 1 Department of Genetics, Nicolaus Copernicus University in Toruń, Lwowska 1, 87-100 Toruń, Poland; 2 Centre for Modern Interdisciplinary Technologies, Nicolaus Copernicus University in Toruń, Wileńska 4, 87-100 Toruń, Poland; 3 The Franciszek Górski Institute of Plant Physiology, Polish Academy of Sciences, Niezapominajek 21, 30-239 Kraków, Poland; 4 Department of Geobotany and Landscape Planning, Nicolaus Copernicus University in Toruń, Lwowska 1, 87-100 Toruń, Poland

**Keywords:** Transcription, Plant sciences

## Abstract

Drought is one of the major threats to food security. Among several mechanisms involved in plant stress tolerance, one protein family—the plant metallothioneins (MTs)—shows great promise for enhancing drought resistance. Plant metallothioneins in oat (*Avena sativa* L.) have not yet been deeply analysed, and the literature lacks a comprehensive study of the whole family of plant MTs in response to drought. In this study, we showed that the number and nature of *cis*-elements linked with stress response in promoters of *AsMTs1–3* differed depending on the MT type. Drought stress in oat plants caused an increase in the expression of *AsMT2* and *AsMT3* and a decrease in the expression of *AsMT1* compared to well-watered plants. Moreover, the low values of relative water content, water use efficiency, net photosynthesis (*P*_*N*_), transpiration (*E*), stomatal conductance (*g*_*s*_), chlorophyll *a*, and carotenoid were accompanied by high levels of electrolyte leakage, internal CO_2_ concentration (*C*_*i*_) and abscisic acid content, and high activity of antioxidants enzymes in plants under drought stress. The present study puts forward the idea that *AsMTs* are crucial for oat response to drought stress not only by regulating antioxidant activity but also by changing the plant water regime and photosynthesis. Our results support the hypothesis that structural differences among types of plant MTs reflect their diversified physiological roles.

## Introduction

Anthropogenic activities have raised the level of CO_2_ and other greenhouse gasses in the atmosphere by 50% since the eighteenth century. As a result, Earth’s temperature is rising and rainfall patterns are changing^1^. Studies show that the Earth’s surface temperature is expected to exceed the limit of 2 °C above pre-industrial levels (1850–1900) by the end of the twenty-first century^2^. As Earth warms, incidents of drought will be longer and more severe^3^.

Water deficit is one of the crucial factors limiting plant productivity and thus threatening food security. Agricultural or ecological drought occurs when water demand exceeds supply^1^. When subjected to drought stress, plants adopt one or more of the four main survival strategies, i.e. (1) drought avoidance, (2) drought escape, (3) drought resistance, and (4) drought recovery. To avoid drought, plants reduce water loss by partial stomatal closure, increased leaf wax accumulation, and leaf rolling^4^. Moreover, a well-developed root system enhances water uptake ability. Drought escape is the natural adjustment of the growth period and life cycle of a plant or artificial changes in planting time by farmers in order to decrease the possible harmful effects of drought. Drought resistance and recovery are the ability of plants to sustain a certain level of physiological activities under drought-stress conditions and then resume growth in non-drought conditions^4^.

In response to drought, several mechanisms are activated in plant cells. To maintain cell turgor pressure, plants produce osmolytes such as proline, soluble sugars, spermine, and betaine^5–8^. In drought conditions, the production of abscisic acid (ABA) is induced. ABA is a stress-response phytohormone and functions as a crucial signal molecule in plant response to drought^9^. ABA triggers a range of physiological processes including induction of stomatal closure, modulation of root development, and inhibition of plant growth^10,11^. Several lines of evidence have shown that drought-responsive genes can be classified into two groups: ABA-dependent and ABA-independent^12,13^. Uno et al.^12^ showed that many ABA-dependent drought-related genes possess an ABA-responsive *cis*-element (ABRE) in the promoter region. On the other hand, genes induced by drought that are not induced by ABA possess other *cis*-elements including drought-responsive elements (DRE) and C-repeat (CTR)^14^.

Drought stress leads to increased production of reactive oxygen species (ROS). This causes oxidative damage to lipids, proteins, and DNA, which can lead to cell death^15^. Plants possess enzymatic and non-enzymatic antioxidant systems. The enzymatic antioxidant system consists of superoxide dismutase (SOD), which catalyses the dismutation of superoxide anion radical (O_2_^·−^) to hydrogen peroxide (H_2_O_2_) and oxygen (O_2_). Hydrogen peroxide is converted to water and oxygen by ascorbate peroxidase (PX) in the presence of a reducing agent such as ascorbic acid or by catalase (CAT)^16^. The non-enzymatic antioxidants include various reducing compounds, such as tocopherols, glutathione, flavonoids, carotenoids, and ascorbic acid. Moreover, under drought, plants accumulate phenolic compounds, which can function as sources of electrons and protons for reactive oxygen species^17–19^_._

Metallothioneins (MTs) are a family of small cysteine-rich proteins present in eukaryotes^20^ and some prokaryotes^21^. In plants, MTs (pMTs) are divided into four types depending on the amino-acid sequence, i.e. pMTs belonging to type 1 (MT1) have 12 cysteine residues, type-2 MTs (MT2) contain 14 cysteines, type-3 MTs (MT3) have ten cysteines, and type-4 pMTs (MT4) contain 17 cysteines^22,23^. MTs have been shown to bind a variety of heavy metal ions (in particular Cu^+^, Zn^2+^, and Cd^2+^) via thiol groups of cysteine residues^24–30^. Some plant MTs have one or more histidine residues, which can also play a role in binding metal ions^31,32^. The expression of p*MT* genes is spatiotemporal and induced by various stimuli, including drought, which suggests that pMTs have a role that goes beyond the maintenance of micronutrient homeostasis and toxic metal detoxication^33,34^. Several lines of evidence have shown that thiol groups of pMTs are powerful antioxidants and can protect plants from oxidative stress^33^. Moreover, MTs can, by binding Cu^+^ ions, stop the Fenton reaction^35–38^.

One of the most cultivated cereals worldwide is oat (*Avena sativa* L.)^39^. This plant is mostly used as livestock feed, but every year it is increasing in popularity as human food. Oat has many nutritional benefits due to its high levels of calcium, soluble fibre, oil, and protein^40–42^. Oat is also a popular and proven-to-work ingredient in various skincare cosmetics. There are some clear indicators that pMTs play a role in drought tolerance, i.e. the increased expression of pMTs in water-limiting conditions has been observed for various plant species^43–46^, and the expression of several pMTs is regulated by ABA^47–49^. We propound the hypothesis that certain pMTs might play essential roles in plant drought resistance. The literature information concerning pMTs and drought is rather scarce and usually limited to only one type of pMT. Therefore, we aimed to analyse and compare the possible roles that MTs of types 1–3 play in response to drought in single plant species. We chose economically important oat since the growth and yield of this crop plant are significantly limited by drought stress^15^. Moreover, we examined the physiological and biochemical parameters reflecting the water regime, photosynthesis efficiency, and antioxidant activity of oat plants subjected to drought stress. The knowledge generated in this study that allows us to gain deeper insight into the mechanisms of oat response to drought stress may enable us to obtain oat varieties more tolerant to drought stress and to reduce yield losses.

## Results and discussion

*A. sativa* is a crop of increasing interest as it is well-adapted to a wide range of soil types. It can perform better than other small-grain cereals on marginal soils. However, oat is sensitive to hot, dry weather, and hence, in several regions of the world, drought is the main factor limiting the yield of oat^39^. To succeed in breeding programmes, the selection of plants with complex traits such as drought resistance should be based upon a comprehensive understanding of innate tolerance mechanisms^50^. In the face of global warming and a growing world population, an understanding of the cellular mechanisms underlying drought tolerance seems to be crucial for food security.

Since their discovery in wheat germs in 1987^51^, plant metallothioneins have been linked with various physiological roles including micronutrient homeostasis^52^, toxic metal detoxication^53^, reactive oxygen species scavenging^54^, senescence^55^, and stress response^56^. Plant MTs have been investigated in various plants such as *Arabidopsis thaliana* (L.) Heynh.^57–60^, *Nicotiana tabacum* L.^61^, *Ipomoea nil* (L.) Roth^22^, *Brassica napus* L.^24,38,62^, *Cucumis sativus* L.^63^, *Oryza sativa* L.^64^, and *Zea mays* L.^65^. However, only one report on *A. sativa* metallothioneins has been published so far^66^. This may be because oat is an allohexaploid species (2*n* = 6× = 42, AACCDD) with a large genome (12.5 Gb), which makes it difficult to work with on the gene level^67^. Beginning in 2016, attempts were made to sequence the oat genome^68^, and in March 2022 the oat genome was published^69^. This will significantly accelerate the research on oat.

### In silico analyses

Plant metallothioneins have been divided into four types based on the number and arrangement of cysteine residues. In all those angiosperm genomes analysed to date, all four types of pMTs are present. There is no clear picture of the possible physiological roles of each type of pMT. It is possible that there is no single unifying role of plant metallothioneins and that one type fulfils different functions depending on the stage of plant development, plant organ, and environmental conditions^70^. Type 4 pMTs are the best-known type of pMTs. This type of pMTs was excluded from this study because pMT4s are seed-specific proteins and in most analysed up-to-date species the expression of this type of pMTs is restricted to developing and mature seeds.

**Figure 1:**
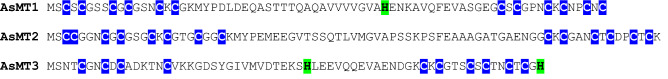
Sequences of putative AsMT1-3 proteins. Cysteines are highlighted in blue, histidines are highlighted in green.

The putative amino acid sequences of oat metallothioneins analysed in this study have a high cysteine content. The number and arrangement of cysteines allow oat MTs to be classified into three types—AsMT1, AsMT2, and AsMT3 (Table 1). The predicted proteins were 64 (AsMT3) to 79 (AsMT2) amino acids long, and the molecular masses ranged from 6814.56 to 7594.64 kDa. The isoelectric point (pI) of AsMT1–3 was similar for all three proteins, and it ranged from 4.85 to 5.10 pI (Table 1). AsMT1 shares the highest homology with MT1 from *Festuca rubra* (87.93%, O24528.1) and MT1 from *Hordeum vulgare* (78.67%, CAD54078.1), AsMT2 is most similar to MT2a from *Lolium rigidum* (84.51%, XP_047054761.1) and MT2 from *Poa secunda* (84.72%, AAK38824.1), and AsMT3 is most similar to MT3 from *Oryza coarctata* (73.44%, AAF68995.1) and MT3 from *Carica papaya* (70.77%, XP_021894753.1). Comparison of the oat MT sequences cloned by our group (Bingo cultivar) with the sequences deposited in the PanOat database revealed 100% identity for AsMT1, AsMT2 and AsMT3 (AVESA.00001b.r3.7Cg0001922, AVESA.00001b.r3.1Cg0000164 and AVESA.00001b.r1.3Ag0000786, respectively). Similarly to MTs from other plant species, AsMT1–3 had two Cys-rich domains separated by one Cys-free stretch. Two His residues were present in AsMT3 and one in AsMT1 (Fig. 1). His residues are involved in the binding of metal ions by MTs, which has been confirmed for bacterial metallothioneins^71^ and type 4 plant MTs^32^. As in AsMT3, it is common for type-3 pMTs to have one or more His residues: one His residue located at the C-terminus of the protein, and a second His residue located in the spacer region of MT. In AsMT1, His residue is located in the middle of the Cys-free region, which is rather uncommon for this type of pMT^70^. The potential involvement of histidines in metal binding has been suggested also for type 3 pMTs^31^.

**Table 1:** Characteristics of putative AsMT1–3 proteins.

Parameter	AsMT1	AsMT2	AsMT3
Length (aa)	72	79	64
Cys content (%)	16.7	17.7	15.6
pI	5.00	5.10	4.85
Mw (kD)	7289.20	7594.64	6814.56

**Figure 2:**
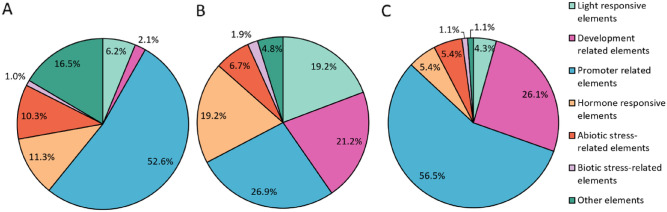
Pie charts depict frequencies of putative *cis*-regulatory elements in *A. sativa* L. *AsMT1* (**A**), *AsMT2* (**B**) and *AsMT3* (**C**) gene promoters. *Cis*-regulatory elements were categorised into seven types according to their predicted functions.

Promoter analysis is a powerful tool that can provide an insight into the regulatory mechanisms of genes of interest. Moreover, studies on *cis*-regulatory elements (CREs) provide a foundation for future experiments^54,72,73^. Regulatory elements play a crucial role in plant responses to various stresses, including drought stress^74^. There are not many studies comparing promoters of different types of *MTs* in one plant species^64,65,72^. Our analyses of oat metallothionein promoters revealed the presence of several *cis*-acting elements involved in response to light, phytohormones, biotic and abiotic stress, and plant development (Fig. 2, Supplementary Table S1). CREs are not distributed equally among promoters of oat *MT*s. A similar observation was made for MT promoters of *B. napus*, *N. tabacum*, and *Z. mays*^61,65,72^. The highest number of CREs was found in *AsMT2* (104) and the lowest in *AsMT3* (92), whereas *AsMT1* promoter contains 97 CREs (Supplementary Table S2). Elements involved in abscisic acid (ABA), jasmonic acid (MeJA), gibberellin, and auxin response were found, of which the first two were the most numerous. ABA-responsive *cis*-elements, also called ABRE, were present in all oat metallothionein promoters: the promoter of *AsMT2* had seven ABRE elements, *AsMT1* had four and *AsMT3* had only one (Supplementary Table S1). In *N. tabacum*, ABRE elements were the most abundant regulatory motifs and were present in all 12 *NtMT* promoters^61^. The promoter of *AsMT2* had the highest number of elements involved in light response (20), while *AsMT1* and *AsMT3* had six and four light-responsive elements, respectively (Supplementary Table S2). *Cis*-regulatory sequences related to the response to light have been identified in *MT* promoters in other plants, e.g. in *MT2* promoter of *L. esculentum*^75^*. AtMT1B* and *AtMT1C* in *A. thaliana*, *OsMT1F*, *OsMT2A*, and *OsMT2B* in *O. sativa*^72^, *EgMT3A* and *EgMT3B* in *Elaeis guineensis*^76^, *CgMT1* in *Casuarina glauca*^77^, and *BrMT1* and *BrMT2* in *B. rapa*^78^. The analysed promoters also contained several development-related elements, i.e. seven elements in *AsMT2* and ten elements in *AsMT3*. Interestingly, *AsMT1* had only two development-related CREs (Supplementary Table S1).

Stress responsive elements were numerous in promoters of oat MTs, i.e. *AsMT1* (11.3%), *AsMT2* (8.6%), and *AsMT3* (6.5%) (Fig. 2). Crucially, drought-related CREs were the most common stress-responsive elements among promoters of *AsMT1–3*. *AsMT1* had nine drought-responsive elements, *AsMT2* had six and *AsMT3* had two (Supplementary Table S1). A regulatory element associated with drought response has been identified in the promoter of the rice *OsMT2b* gene^79^. Interestingly, elements involved in response to other stresses, such as fungal elicitor response, wounding response, and anaerobic conditions response were not distributed evenly among oat metallothioneins. In the promoter of *AsMT3*, there were no elements associated with wounding response, however; only in the promoter of this gene CREs involved in the response to fungal elicitors and anaerobic conditions were present (Supplementary Table S1).

Experimental research confirming the functionality of in silico identified CREs in plant MT promoters is scarce. The function of MT promoters has usually been studied using transgenic *A. thaliana* plants, where the promoter was fused with β-glucuronidase (GUS). For example, in the study by Ren and Zhao^79^, a rice type 2 MT promoter was especially induced by wounding, ABA, gibberellin, cytokinin, PEG, cold, hot, NaCl and Zn treatment, and, in that promoter, respective CREs were found. In another, similar study, the promoter of another rice type 2 MT had ABA and metal-responsive CREs, and the application of ABA, Zn, and Cu caused an increase in GUS levels^80^. The promoter of rice type 1 metallothionein has been shown to be responsive to ABA, drought, dark, Zn, Cu, Pb, and Al, and respective CRE motifs have been found in the promoter sequence^81^. In an analysis of type 1 MT promoter from *C. glauca*, CREs involved in the response to metals and wounding were found. The researchers found that the promoter was indeed responsive to wounding, but did not find the responsiveness to metals that the promoter analysis suggested. In transgenic *A. thaliana*, levels of GUS did not increase significantly after Cu, Zn, and Cd treatment, whereas wounding and H_2_O_2_ treatments led to an increase in levels of the reporter gene activity^82^.

### *AsMT1–3* expression in response to drought stress

**Figure 3:**
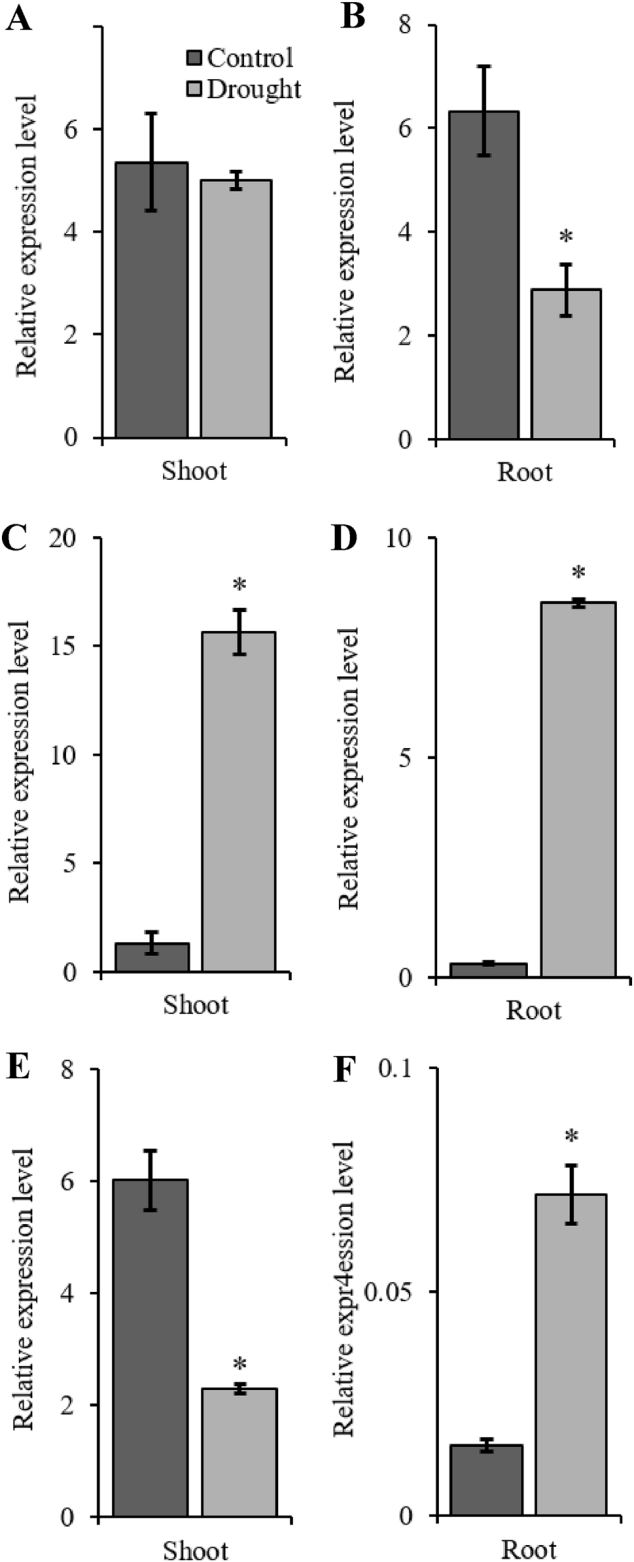
Relative gene expression of *AsMT1* (**A**, **B**), *AsMT2* (**C**, **D**) and *AsMT3* (**E**, **F**) in shoots and roots of oat seedlings in control and drought conditions. *AsMT1-3* genes were quantified with RT-qPCR and normalised using housekeeping gene *EIF4A*. Bars represent mean values ± SE of three replicates. Student’s t-Test, *p* ≤ 0.05.

In a limited number of studies, the upregulation of pMTs in response to drought has been shown, e.g. during drought stress, a higher expression of type 2 *MT* in watermelon^83^ and a three-fold increase in the expression of *MT3* in leaves of buckwheat (*Fagopyrum esculentum* Moench) were observed^84^. Here, the exposure of oat seedlings to drought stress caused significant changes in *AsMT1–3* expression in oat shoots and roots. As mentioned before, in promoters of *AsMTs*, we found elements involved in ABA and drought response, but each *AsMT* promoter had a different number of those elements. In drought-stressed plants, the expression of *AsMT1* in the shoots did not change, and the *AsMT1* expression level in the roots was half that of control plants (Fig. 3A,B). The highest upregulation by drought was observed for *AsMT2*, i.e. it was 12-fold higher in the shoots and 27-fold higher in the roots in comparison to control plants (Fig. 3C,D). The expression of *AsMT3* in the shoots of drought-stressed plants was 2.6 times lower (Fig. 3E), but in the roots of drought-stressed plants a 2.6-fold increase was detected (Fig. 3F). Interestingly, the total number of ABA-responsive and drought-responsive CREs in the promoter regions of *AsMT1* and *AsMT2* is the same (13), but *AsMT2* had more ABA-responsive elements than drought-responsive elements, whereas the opposite is observed for *AsMT1* (Supplementary Table S1). *AsMT3* has the fewest drought-related and ABA-responsive *cis*-elements. These results indicate different roles of oat MT1–3 in drought response and suggest that the expression of oat *MT*s in drought-stressed plants is regulated via the ABA-dependent pathway. Previous studies have revealed that the expression levels of some *MT* genes, such as *OsMT1a*^44^, *OsMT2b*^85^, *GhMT3a*^43^, and *BrMT1*^78^, are increased by ABA treatment, while the transcription levels of *BrMT2* and *BrMT3* were downregulated^78^.

Contrary to our results, Jaiswal et al.^86^ showed that, under drought stress, the expression of genes of *MT*2 and *MT3* from guar (*Cyamopsis tetragonoloba* L.) was unchanged in roots or shoots, and the *MT1* gene was upregulated in both organs of the plant. Exposing *Citrullus lanatus* (Thunb.) Mansf. to drought stress resulted in increased expression of 32 genes, one of which was homologous to *Lycopersicon esculentum* L. *MT2*^83^. Overexpression of metallothioneins confers drought stress tolerance in plants. For example, drought-stressed *A. thaliana* L. plants overexpressing type 1 metallothionein from chickpea had longer roots, higher biomass, and higher levels of enzymatic and non-enzymatic antioxidants in comparison to WT^87^. Similar results were obtained for *A. thaliana* L. plants overexpressing the *MT2A* gene from date palm^88^ and *OsMT3-a* from rice^89^. The regulation of *MT* genes expression in response to stress is multi-dimensional. Our results confirm the hypothesis that different types of MTs act differently and have different functions in plant cells. MTs exhibit a strong antioxidant property against oxidative damage via the neutralization of O_2_^·−^ and enhanced H_2_O_2_ scavenging ability^83,90,91^. According to a study done by Li et al.^90^, overexpression of *MT* genes can significantly improve drought tolerance and is accompanied by elevated antioxidant enzyme activities, supporting the view that the MTs are involved in the ROS scavenging pathway.

### Water status and photosynthetic efficiency of oat seedlings under soil drought

Water deficiency is an important factor affecting the growth and yield of plants subjected to drought. During the drought period, disturbances of many metabolic processes such as photosynthesis are observed^92^. The ability to retain stability of cell membrane under drought stress is one of the key physiological indices widely used to evaluate the drought tolerance of plants^93^. Measurements of relative water content (RWC), water loss (WL), electrolyte leakage (EL), and photosynthetic water use efficiency (WUE) are parameters frequently used as a selection test for the assessment of plant cultivar tolerance to various stresses^94–97^.

**Figure 4:**
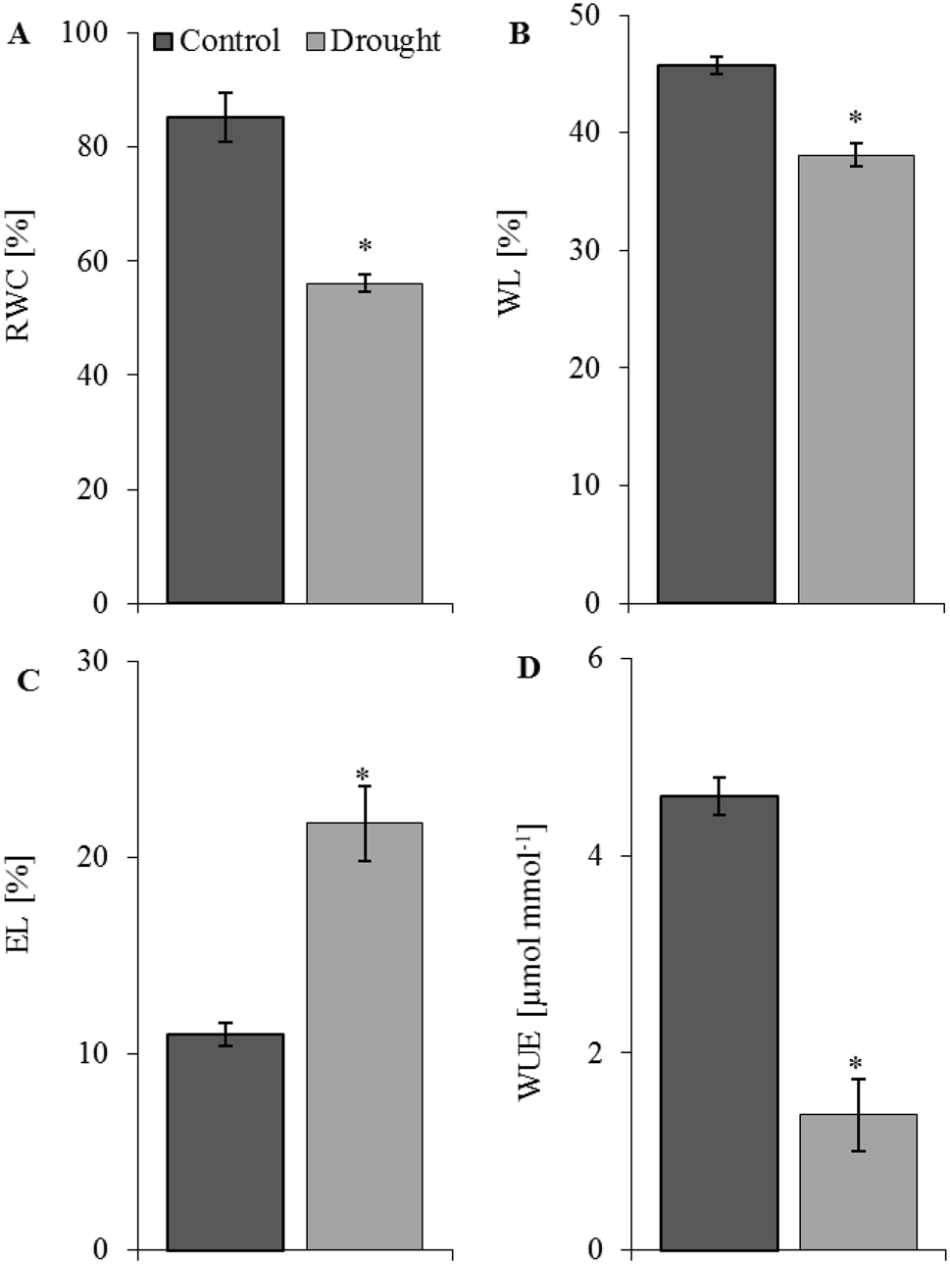
Water status: relative water content—RWC (**A**), water loss—WL (**B**), electrolyte leakage—EL (**C**), and photosynthetic water use efficiency—WUE (**D**) in shoots of oat seedlings in control and drought conditions. Bars represent mean values ± SE. Student’s t-Test, *p* ≤ 0.05.

According to Hsiao^96^, the level of RWC drop corresponds to the severity of the water stress. In our study, drought caused a significant decrease in leaf RWC (Fig. 4A), i.e. from 85.3% in well-watered plants to 56.1% in drought-treated plants. In response to drought, a significant decrease in WL (Fig. 4B) and a significant increase in EL (Fig. 4C) were observed. Both parameters show the loss of cell membrane permeability and are changed under many stresses^95^. The photosynthetic water use efficiency (WUE), defined as the ratio of carbon assimilation to transpiration, was considerably lower in drought-stressed plants (1.4 µmol mmol^−1^) in comparison to well-watered plants (4.6 µmol mmol^−1^) (Fig. 4D). WUE is controlled by synchronising the relation between carbon assimilation and water intake, which is a significant strategy used by plants to survive drought. In the study by Liang et al.^98^, WUE and each of the gas exchange parameters of tomato leaves decreased in response to low levels of soil moisture. It is a common phenomenon that drought limits plant growth by reducing the photosynthetic rate. The key reasons for decreased photosynthesis are stomatal closure caused by decreased CO_2_ levels and reduced photosynthetic activity in the mesophyll^98^. At the beginning of drought stress, the stomata close first to reduce water transpiration, and as a result, the level of CO_2_ in the leaves decreases. When the decrease in net photosynthesis (P_*N*_) as a result of drought is accompanied by increased (or unchanged) internal CO_2_ concentration (*Ci*), non-stomatal factors are the main cause of reduced photosynthetic rate; meanwhile, when decreased *P*_*N*_ is accompanied by decreased *Ci*, stomatal factors are the main cause.

**Figure 5:**
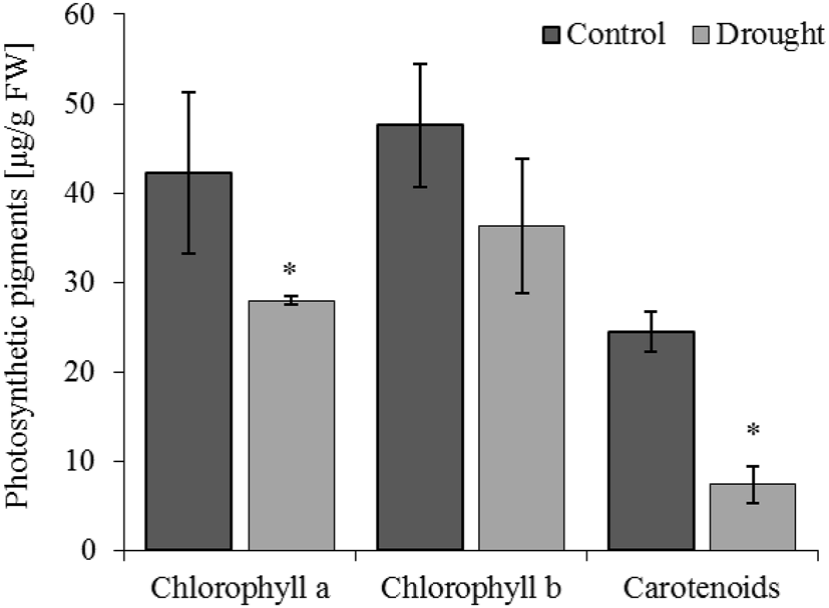
Photosynthetic pigments (chlorophyll *a*, chlorophyll *b* and carotenoids) in shoots of oat seedlings in control and drought conditions. Bars represent mean values ± SE. Student’s t-Test, *p* ≤ 0.05.

**Figure 6:**
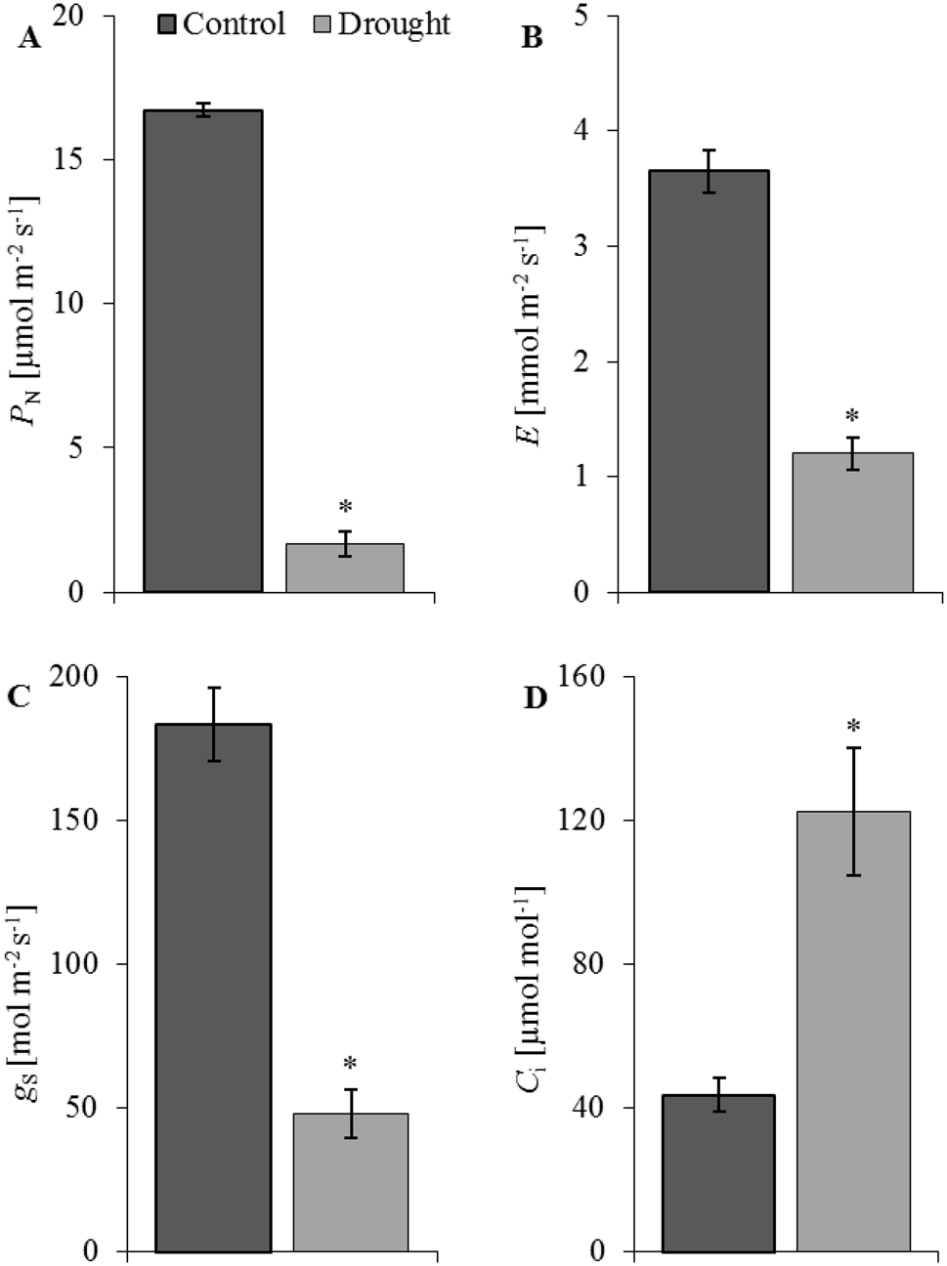
Leaf gas-exchange parameters: net photosynthesis—*P*_*N*_ (**A**), transpiration—*E* (**B**), stomatal conductance—*g*_*s*_ (**C**), and internal CO_2_ concentration—*C*_*i*_ (**D**) in shoots of oat seedlings in control and drought conditions. Bars represent mean values ± SE. Student’s t-Test, *p* ≤ 0.05.

Usually, under moderate and severe drought stress, the *Ci* gradually increases as the *P*_*N*_ and stomatal conductance (*g*_*s*_) decrease. This indicates that non-stomatal restriction is the main factor of the decrease in the photosynthetic rate as the drought stress extends, which could lead to damage to the chloroplast structure^99^. In our study, drought significantly reduced the content of chlorophyll *a* and carotenoids in oat leaves (Fig. 5). Moreover, the net photosynthesis (*P*_*N*_), transpiration (*E*), and stomatal conductance (*g*_*s*_) drastically decrease in drought-treated plants (Fig. 6A–C). These changes accompany a substantial increase in internal CO_2_ concentration (*C*_*i*_) (D) in oat leaves under drought (Fig. 6D). These observations suggest that nonstomatal restriction was accountable for reduced photosynthesis in oat leaves. As described by Zhao et al.^100^ and Zhang et al.^99^, *P*_*N*_, *E*, *g*_*s*_ decreased significantly and were strictly associated with the degree and duration of drought stress in *Avena nuda* L. and *A. sativa*. A drought-prompted decrease in the photosynthetic activity of wheat leaves was also reported by Todorova et al.^101^. A large decrease in *P*_*N*_ and *g*_*s*_ has been observed in drought-stressed oat plants in comparison to control plants, while a lower decrease has been observed for *E* and WUE. Also, *P*_*N*_ rate has been closely related to chlorophyll loss^102^ and all photosynthetic pigments, as well as the disruption or loss of thylakoid membranes^103^.

### The activity of the antioxidant system of oat seedlings

**Figure 7:**
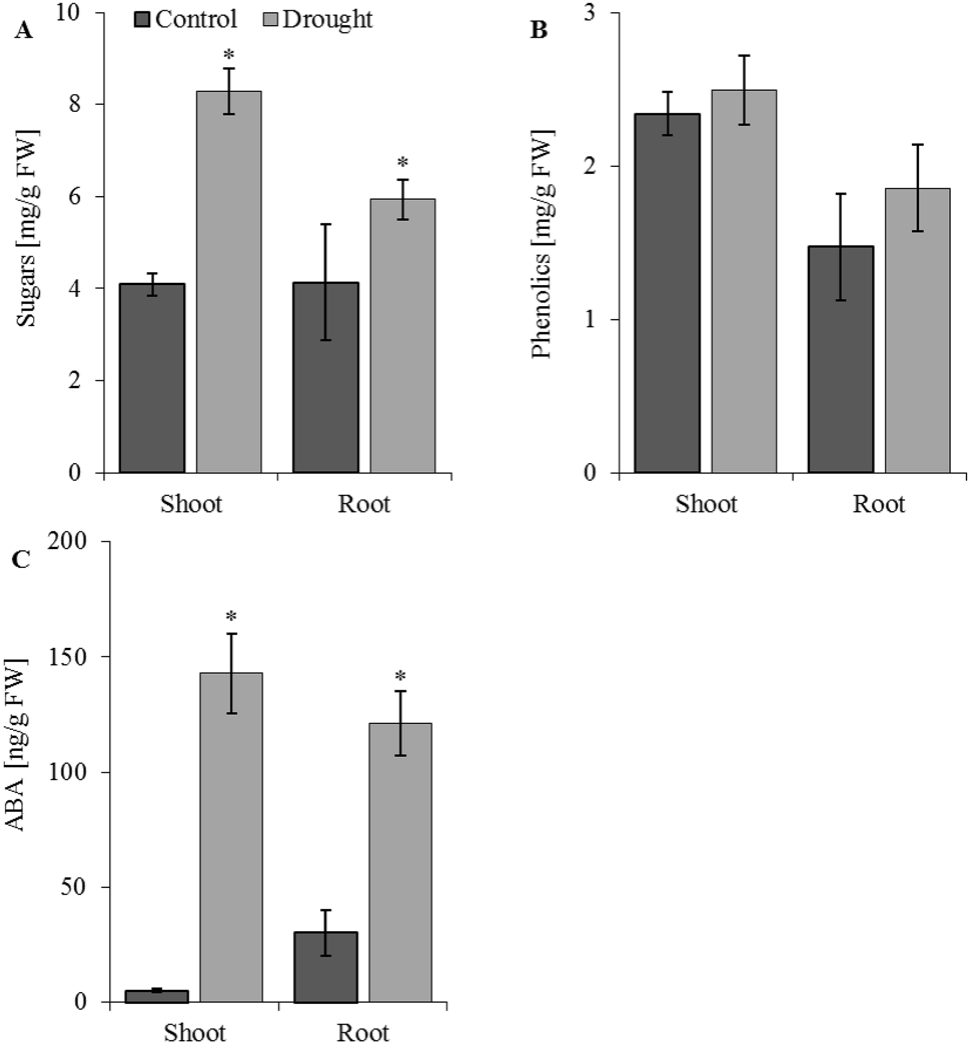
Concentration of sugars (**A**), phenolics (**B**) and abscisic acid (ABA) (**C**) in shoots and roots of oat seedlings in control and drought conditions. Bars represent mean values ± SE. Student’s t-Test, *p* ≤ 0.05.

Biochemical responses of crops associated with tolerance to drought are linked to changes in metabolic pathways, leading to the production of, e.g., sugars and phenolic compounds^104^. These metabolites mainly act as osmolytes, which reduce cellular dehydration and participate in the stabilization of enzymes and cellular membranes^105^. In our study, soil drought markedly increased the content of soluble sugars in roots and shoots of oat seedlings (Fig. 7A), whereas the amount of phenolic compounds was unaffected (Fig. 7B). As reported by Arabzadeh^106^, the accumulation of sugars by plants enhances water-holding capacity in cells and can thus reduce drought stress via regulation of the plant's osmotic potential.

**Figure 8:**
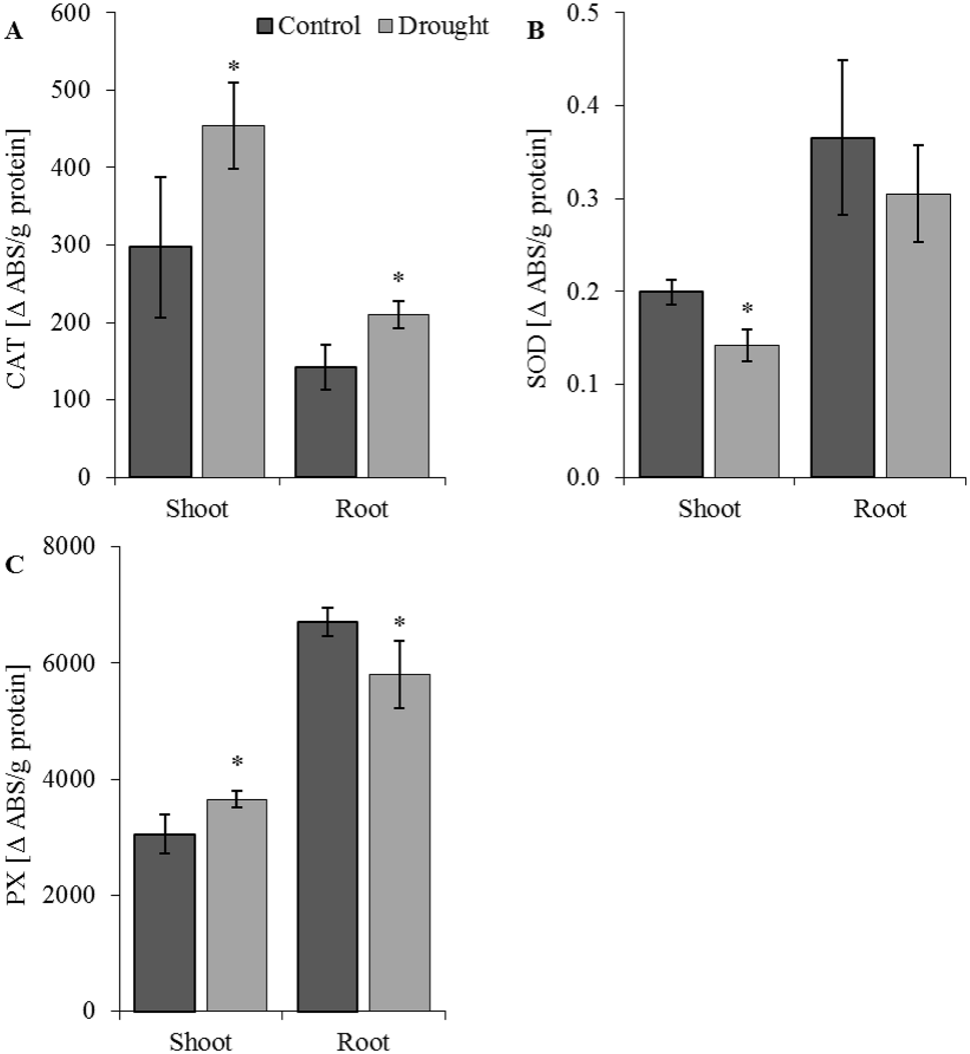
Activity of antioxidant enzymes: catalase—CAT (**A**), superoxide dismutase—SOD (**B**), and peroxidase—PX (**C**) in shoots and roots of oat seedlings in control and drought conditions. Bars represent mean values ± SE. Student’s t-Test, *p* ≤ 0.05.

An oxidative burst commonly occurs in response to various stress conditions^107^. The question of whether plant MTs are general stress proteins because of their potential to scavenge radicals^108^ or whether they are involved in response to some limited stress conditions is still open. Figure 8 shows the activities of antioxidant enzymes: catalase (CAT), superoxide dismutase (SOD), and peroxidase (PX) in shoots and roots of oat plants in response to drought. Compared to well-watered plants, CAT activity increases significantly after drought treatment in oat roots and shoots (Fig. 8A), while SOD activity decreases (Fig. 8B). PX activity was significantly higher in shoots and lower in roots of drought-treated plants in comparison to control plants (Fig. 8C). In our previous study^66^, in oat seedlings subjected to osmotic stress, CAT and PX had the highest activity in the treated plants and SOD had the lowest. A similar observation was reported by Chakraborty and Pradhan^109^ in *Triticum aestivum* L., where SOD activity showed a general decline in activity and the activity of PX increased greatly during water deficit. This is in line with Gratão et al.^110^, who hypothesised that SOD acts as the first line of defence against. H_2_O_2_ produced by SOD is then metabolized by the next enzyme, CAT. In our study we also observed a slight, but insignificant, increase in phenolic compounds in both roots and shoots of drought-treated plants (Fig. 7B). Soluble sugars and phenolics eliminate H_2_O_2_, and thus reduce the harmful effects of oxidative stress^111^.

Many studies emphasize the well-established role of ABA in physiological processes and acclimation to abiotic stresses, thereby assigning it the role of a positive regulator of plant drought resistance^112,113^. Here, high levels of ABA were observed in shoots (142.8 ng/g FW) and roots (121.1 ng/g FW) of drought-treated plants comparing to control (5.0 ng/g FW and 30.2 ng/g FW, respectively) (Fig. 7C). In research by Peltonen-Sainio and Mäkelä^114^ on 19 oat cultivars, it was determined that drought stress significantly increased the accumulation of ABA, whereas *C*_*i*_ and RWC decreased due to water deficit. It is known that ABA is a key regulator of abiotic stress resistance in plants. It mediates many stress-responsive genes, including genes regulating the efficiency of photosynthesis. ABA-induced stress tolerance is partly associated with the action of antioxidant systems, which protects plant cells from oxidative damage^14^.

### Correlations between gene expressions, water status and stress responses

**Figure 9:**
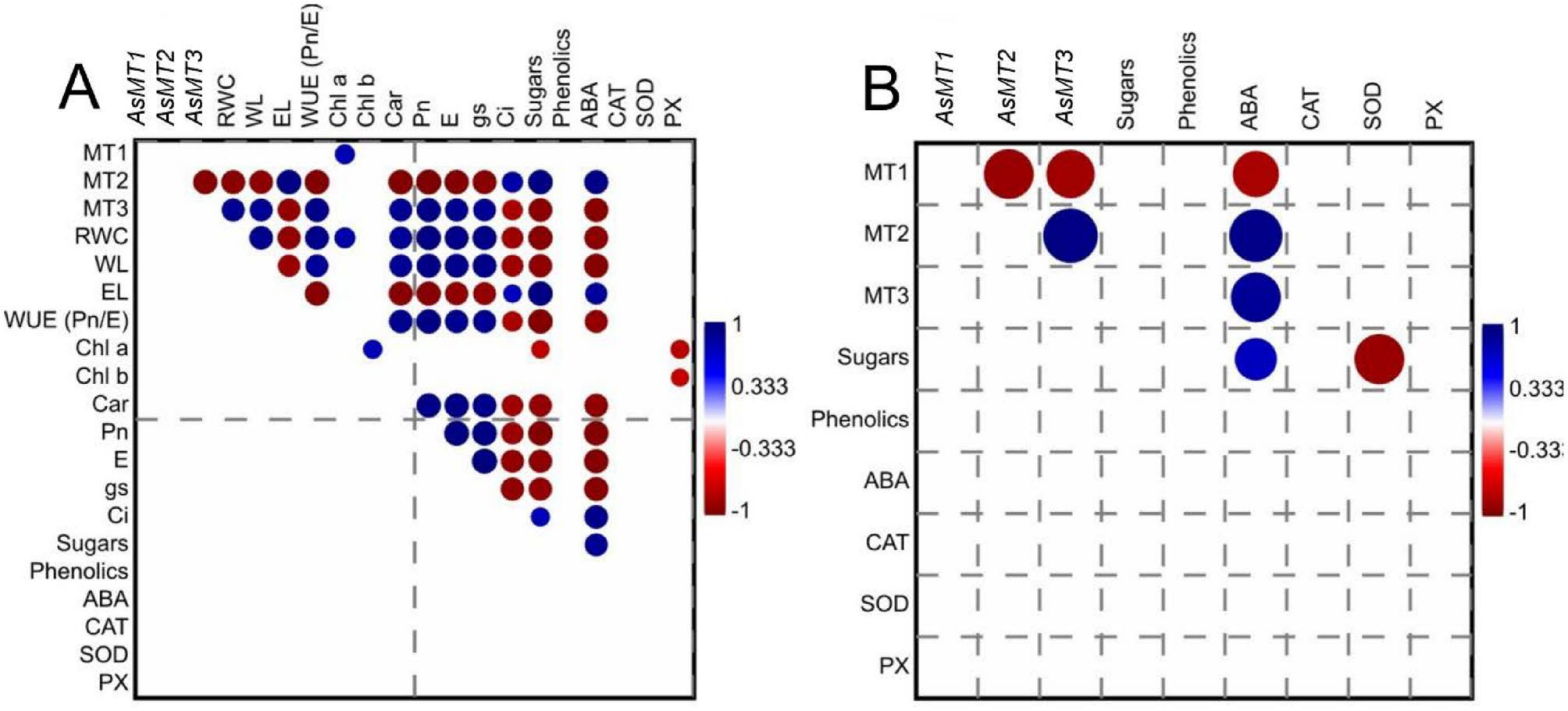
Pearson correlations between *AsMT* gene expressions and measured plant traits in shoots (**A**) and in roots (**B**). Only significant relations are demonstrated (*p* < 0.05). Abbreviations: *AsMT1*—oat metallothionein type 1, *AsMT2*—oat metallothionein type 2, *AsMT3*—oat metallothionein type 3, RWC—relative water content, WL—water loss, EL—electrolyte leakage, WUE—photosynthetic water use efficiency, *Chl* a—chlorophyll a, *Chl* b—chlorophyll b, Car—carotenoids, Pn—net photosynthesis, E—transpiration, gs—stomatal conductance, Ci—internal CO_2_ concentration, ABA—abscisic acid, CAT—catalase activity, SOD—superoxide dismutase activity, PX—peroxidase activity.

In our study, the expressions of *AsMT2* and *AsMT3* were significantly negatively correlated to each other in shoots but positively correlated in roots. The expression of *AsMT1* was independent in shoots but negatively correlated with the expression of *AsMT2* and *AsMT3* in roots (Fig. 9). *AsMT2* expression was positively correlated with EL, Ci, sugars, and ABA in shoots, conversely to *AsMT3*, which was negatively correlated with these parameters. A negative correlation between the expression of *AsMT2* and RWC, WL, WUE, Car, P_*N*_, E, and *g*_*s*_ was observed in shoots. Inverse correlations were observed for *AsMT3* expression and the mentioned parameters. In both shoots and roots, the expression of *AsMT2* was positively correlated with ABA. This might confirm that *AsMT2* is involved in oat response to drought and is regulated via an ABA-dependent pathway. The expression of *AsMT3* negatively correlated with ABA in shoots, but in roots the correlation was positive. Interestingly, *AsMT3* had significantly fewer ABA-responsive CREs than *AsMT1* and *AsMT2*. The expression level of *AsMT1* was positively correlated with chlorophyll *a* content in shoots and negatively correlated with ABA in roots (Fig. 9). Interestingly, no correlation between *AsMTs* expression and antioxidant enzymes was observed. A negative correlation was observed between PX activity and levels of chlorophyll in oat shoots. In oat roots, a negative correlation was observed between SOD activity and levels of sugars.

Drought stress is extensively investigated in plants of industrial importance, including oat. However, still little is known about the molecular mechanisms underlying oat’s tolerance of or susceptibility to drought^50^. Thus, it is necessary to conduct further physiological and molecular research concerning the responses of oat to drought stresses. Plant MTs seem to participate in plant drought tolerance, but the exact pathways are still unclear, and more in-depth research is needed. Our results showed that oat metallothioneins type 1–3 have different roles in plant cells in response to drought stress. During drought stress in oat plants, the efficiency of photosynthesis decreased and the content of ABA significantly increased. We hypothesised that the expression of *AsMT2* was induced via ABA in drought-stressed plants. Metallothioneins together with sugars and antioxidant enzymes (CAT and PX) protect cells from a high level of ROS. We propound the hypothesis that a higher amount of MTs is necessary to provide elevated levels of zinc in cells. Zinc is a crucial cofactor of several enzymes and structural element of countless transcription factors. Moreover, prolonged stress leads to the activation of apoptosis, which is also regulated by Zn ions. Moreover, MTs are crucial for the translocation of zinc and possibly other metal ions to different parts of plants^115^.

In conclusion, the conducted research provides important new information on the response of plants to stress mediated by metallothioneins. This knowledge about the role of AsMTs in drought stress response will enable the creation of plants via conventional or transgenic breeding that will be resistant to stresses, including drought. This will allow for greater yield from crops even in adverse environmental conditions.

## Materials and methods

### In silico analyses of promoters of *A. sativa* L. *MT* genes

We have previously cloned three oat metallothionein partial cDNA sequences^66^. Since then, the genome assembly of *A. sativa* has been published in the PanOat database (https://wheat.pw.usda.gov/GG3/PanOat)^116^. For each *AsMT* gene, a 1500-bp-long fragment of genomic DNA upstream of the start codon was retrieved from the PanOat database^116^. The promoters were analysed using the PlantCARE database^117^. Molecular masses and pI of putative oat MT proteins were calculated using the Compute pI/Mw tool (ExPaSy)^118^.

### Plant material

**Figure 10:**
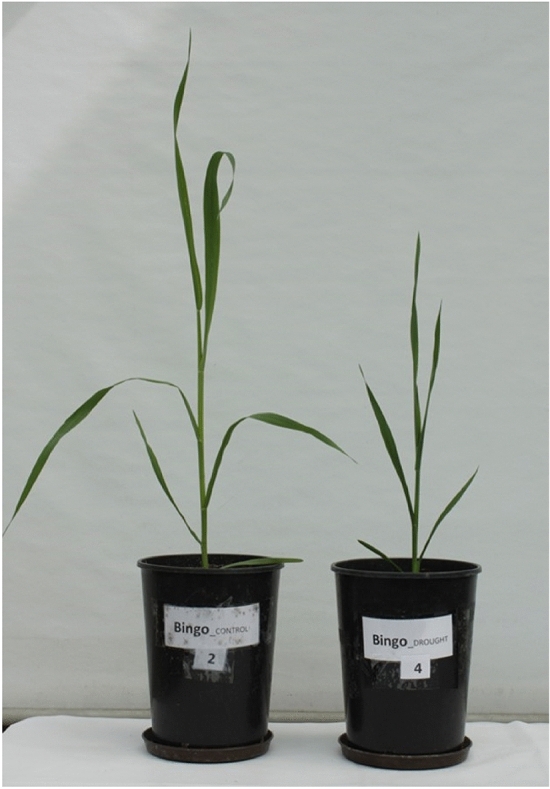
Oat (*Avena sativa* L.) cv. Bingo at day 14 of drought treatment.

Grains of oat cv. Bingo, purchased from Plant Breeding Strzelce Ltd., PBAI Group, Strzelce, Łódź Voivodeship, Poland, were sown individually to 3-dm^3^ pots filled with a mixture of soil and sand (3/1 v/v). Plants were grown at 25 °C under a 16-h photoperiod and 800 μmol (hν) m^2^ s^−1^ PAR. Drought stress was induced by the cessation of watering the soil when the plants reached the four-leaf stage. The degree of soil moisture was determined by the gravimetric method and set at 70% field water capacity (FWC) for control conditions and 20% FWC for drought conditions. After 14 days, leaves and roots of control and drought-treated plants were collected (Fig. 10). The authors confirm that all methods used were performed in accordance with the relevant guidelines and legislation.

### Isolation of nucleic acid and analysis of *AsMT1–3* expression in response to drought stress

The oat plants were washed several times in nuclease-free water. Shoots and roots were ground separately in liquid nitrogen. Total RNA was isolated using RNeasy Plant Mini Kit (QIAGEN, Germany) according to the manufacturer’s protocol. The quality and quantity of the extracted RNA were checked by agarose gel electrophoresis and by spectrophotometric measurement using a NanoDrop Lite Spectrophotometer (Thermo Fisher Scientific, USA). To remove any DNA contamination from RNA samples, 1.5 μg of total RNA was treated with 1 U of DNase I (Thermo Fisher Scientific, US) and incubated at 37 °C for 30 min. The reaction was stopped by the addition of 1 μL 50 mM EDTA and incubation at 65 °C for 10 min. Reverse transcription reaction was performed using a RevertAid Reverse Transcriptase (Thermo Fisher Scientific, US) according to the manufacturer’s protocol using 250 ng oligo (dT)_20_ primer and 200 ng random hexamers.

RT-qPCR was performed in a total volume of 10 μL using a Maxima SYBR Green/ROX qPCR Master Mix (Thermo Fisher Scientific, US)^66^. The reaction mixture contained 4 μL of 10 × diluted cDNA and 0.3 μM gene-specific primers (Table 2). Three replicates were performed for each reaction. The qPCR reaction was conducted in a LightCycler 480 Instrument (Roche, Germany). The thermal cycling conditions were as follows: 95 °C for 10 min for initial denaturation, 40 cycles of 95 °C for 15 s, 60 °C for 30 s, and 72 °C for 30 s^66,119^. Differences in the target gene expression were evaluated by a relative quantification method normalising the data to the reference genes for eukaryotic initiation factor 4A-3 (*EIF4A*) and heterogeneous nuclear ribonucleoprotein 27C (*HNR*)^120^. The fold-change in gene expression was calculated using LightCycler 480 Software (ver. 1.5.1.62).

**Table 2:** Primers used for qPCR reactions.

Primer name	Sequence 5′ → 3′	Product size [bp]	Reference
AsMT1_qPCR_f AsMT1_qPCR_r	CAAACTGCAAGTGCGGGAAG TTGTTCTCATGAGCCACGCC	103	^66^
AsMT2_qPCR_f AsMT2_qPCR_r	CTGCGGAGGGTGCAAGATG AACGATGGCTTGGAAGAGGG	96	^66^
AsMT3_qPCR_f AsMT3_qPCR_r	TCCACCATGTCGAACACCTG TGGCTCTTCTCGGTGTCAAC	107	^66^
EIF4A_f EIF4A_r	TCTCGCAGGATACGGATGTCG TCCATCGCATTGGTCGCTCT	88	^120^
HNR_f HNR_r	ATTGGGTTTGTCACTTTCCGTAG CTTGGAGGGTGTCTCGCATCT	134	^120^

### Biochemical analyses

#### Relative water content (RWC)

RWC was determined in leaves according to Ober et al.^121^. Samples were collected from the second fully developed leaf. RWC was calculated according to the equation: RWC (%) = (Wf − Wd)/(Wt − Wd) × 100, where Wf, Wd and Wt represent fresh weight, dry weight, and turgid weight, respectively. The experiment was repeated three times with five plants.

#### Water loss (WL) test

WL in leaves was determined using Clarke and McCaig’s^122^ method. Plants were grown in a greenhouse under well-watered conditions at 21 °C until the fourth leaf had fully emerged. This leaf was cut and placed in a growth chamber at 20 °C, 50% relative humidity, and continuous light of 250 µmol m^−2^ s^−1^. The mass of leaf was recorded after cutting (0 h), 6 h later, and after drying at 70 °C for 48 h. WL was calculated as water loss per unit of initial water content according to the equation: WL (%) = (FW0 − FW6)/(FW0 − DW), where FW0 and FW6 are fresh weights after cutting and 6 h later, respectively, and DW is the dry weight after drying at 70 °C. The experiment was repeated three times with five plants.

#### Electrolyte leakage (EL)

Three leaf discs (1 cm in diameter) were placed into a plastic tube containing 10 mL of redistilled water. They were shaken (100 rpm) at room temperature and the initial electrolyte leakage (EL0) was measured with a conductivity meter (CI 317, Elmetron, Poland) after 24 h. The tubes with leaves were stored at − 70 °C overnight, shaken after thawing, and then their conductivity, and total content of ions (EL1) were measured. The permeability of cell membranes was represented as a percentage of total electrolyte leakage according to the equation: EL = EL0 × 100/EL1. The experiment was repeated three times with five plants.

#### Leaf gas-exchange parameters

The rate of gas exchange was measured in the fully developed second leaf using a portable CIRAS-2 photosynthesis system (PP System, Hitchin, UK). The rate of net photosynthesis (*P*_*N*_) and transpiration (*E*), stomatal conductance (*g*_*s*_), and internal CO_2_ concentration (*C*_*i*_) were measured between 9:00 and 11:00 a.m. The photosynthetic water use efficiency (WUE) was also expressed as P_*N*_/E. The experiment was repeated three times with five plants. The measurements included three replicates per plant.

#### Photosynthetic pigments content

The 100 mg of leaves was homogenized in 1 mL of 80% ethanol and then centrifuged at 2800 rpm for 10 min. The absorbance of the samples was measured at λ = 470 nm, λ = 648 nm, and λ = 664 nm on a micro-plate reader (Synergy 2, Bio-Tek, Winooski, VT, USA). Concentrations of photosynthetic pigments (chlorophylls *a*, *b* and carotenoids) were determined using a Lichtenthaler and the Wellburn method^123^. The experiment was repeated three times with five plants.

#### Soluble sugar content

The 100 mg of leaves was homogenized in 1 mL of 80% ethanol, then centrifuged at 2800 rpm for 10 min. The amounts of total soluble sugars were estimated by the phenol–sulphuric acid method^124^. Briefly, the supernatant was mixed with 5% phenol and 96% sulphuric acid. The absorbance of the samples was measured spectrophotometrically at λ = 490 nm on a micro-plate reader (Synergy 2, Bio-Tek, Winooski, VT, USA). The amount of soluble sugars was expressed as milligrams of glucose per 100 g of fresh mass (FW) of plant tissue. The experiment was repeated three times with five plants.

#### Total phenolics content

The 100 mg of leaves was homogenized in 1 mL of 80% ethanol, then centrifuged at 2800 rpm for 10 min. To estimate the phenolics content, the supernatant was mixed with 20% Na_2_CO_3_ and Folin–Ciocalteu reagent^125^. The absorbance of samples was measured spectrophotometrically at λ = 760 nm on a micro-plate reader (Synergy 2, Bio-Tek, Winooski, VT, USA). The total phenolic content was calculated as milligrams of chlorogenic acid per gram of FW of plant tissue. The experiment was repeated three times with five plants.

#### Abscisic acid (ABA) content

The leaves were frozen in liquid nitrogen, lyophilised and homogenised. Then, 50 mg of plant material was extracted with a 1-mL mixture of methanol/water/formic acid (15/4/1; v/v/v) according to Dobrev and Kaminek^126^. An internal isotopic standard of ABA was added to each sample. The extract was then centrifuged, the supernatant was collected, and the extraction procedure was repeated. The combined supernatant was dried and reconstituted in 1 mL of 1 M formic acid. This extract was fractionated with SPE columns Oasis MCX 1 cc/30 mg (Waters, Milford, MA, USA). The acidic fraction was eluted from the SPE column with 1 mL methanol, evaporated to dryness, and reconstituted in 50 µL methanol. Samples prepared in this manner were analysed on a Supelco Ascentis RP-Amide HPLC column (Saint Louis, MO, USA) (7.5 cm, 4.6 mm, 2.7 µm). Mobile phases were 0.1% formic acid solution in water (solvent A) and acetonitrile/methanol (1/1) mixture. Gradient elution was applied under the flow rate of 0.5 mL/min. The HPLC apparatus was an Agilent Technologies 1260 equipped with an Agilent Technologies 6410 Triple Quad LC/ MS with ESI (Electrospray Interface, Agilent Technologies, Santa Clara, CA, USA). The two most abundant secondary ions were monitored: abscisic acid (ABA)—m/z 265.2 primary, m/z 229.1, 247.1 secondary; D-ABA (deuterium labelled ABA used as internal standard)—m/z 271.2 primary, m/z 167.1 secondary. Ten-point calibration curves were prepared for the analysed compounds. The experiment was repeated three times with five plants.

#### Antioxidant enzymes activities

The leaves were homogenised with 0.05 M phosphate buffer (pH 7.0) containing 0.1 mM EDTA at 4 °C and centrifuged at 2800 rpm for 10 min. Superoxide dismutase (SOD) activity was assayed according to McCord and Fridovich^127^. The reaction mixture consisted of 0.05 M phosphate buffer, 0.013 mM cytochrome c, 0.1 mM xanthine, 0.024 U per ml xanthine oxidase, and supernatant. Absorbance was measured at λ = 550 nm.

Catalase (CAT) activity was determined according to Aebi^128^. The reaction mixture contained 0.05 M phosphate buffer, 0.1 mM H_2_O_2_, and supernatant. The rate of H_2_O_2_ decomposition was measured at λ = 240 nm.

The activity of peroxidase (PX) was measured by the method of Lűck^129^. The amount of oxidation product of 1% p-phenylenediamine in the presence of 0.03 M H_2_O_2_ was measured at λ = 485 nm.

The reaction kinetics of all enzymes were measured spectrophotometrically using a micro-plate reader (Synergy 2, Bio-Tek, Winooski, VT, USA). Enzyme activities were calculated per milligram of protein measured by Bradford method with bovine serum albumin as a protein standard^130^. The experiment was repeated three times with five plants for each enzyme.

## Statistical analysis

The results are expressed as mean values, and error bars represent standard error (SE). Before statistical assessment, data normality was tested by the Shapiro–Wilk test. The majority of parameters were normally distributed, and statistical analysis of the experimental data was done with the analysis of variance (ANOVA). Student’s t-test (*p* value ≤ 0.05) was applied to determine differences between expression levels of control and drought-stressed plants. To demonstrate relations between measured traits, Pearson correlations were calculated^131^. The programs Past 4.0^132^, STATISTICA 13.0 (Stat-soft, Inc., USA), and RStudio^133^ were applied for calculations.

## Supplementary Information

Supplementary Information.

## Data Availability

The datasets generated during the current study are available from the corresponding author on reasonable request.
